# Case report: Traumatic ventricular aneurysm combining tricuspid valve avulsion in a child: Diagnostic findings and treatment protocols

**DOI:** 10.3389/fcvm.2022.928265

**Published:** 2022-08-23

**Authors:** Dengshen Zhang, Shan Wei, Wenhong Tao, Yuanfeng Liao, Ruihan Xiao, Daxing Liu

**Affiliations:** ^1^Department of Cardiovascular Surgery, Affiliated Hospital of Zunyi Medical University, Zunyi, China; ^2^Department of Ultrasonography, Affiliated Hospital of Zunyi Medical University, Zunyi, China

**Keywords:** traumatic tricuspid regurgitation, aneurysmoplasty, blunt cardiac injury, acquired left ventricular aneurysm, cardiac surgery

## Abstract

This case report is an extremely rare case of a traumatic left ventricular aneurysm in a 3-year-old child who also had tricuspid valve avulsion due to blunt trauma. The diagnostic findings and treatment protocols are discussed to provide a clinical reference.

## Introduction

The left ventricular aneurysm can be categorized as congenital or acquired. The congenital left ventricular aneurysm is a dysplastic heart malformation thataffects approximately 0.04% of the population (1). The most common cause of acquired ventricular aneurysms is myocardial infarction, which can also occur in cardiomyopathy, myocarditis, complications of a cardiovascular procedure, and blunt trauma (2). The most uncommon type is a traumatic ventricular aneurysm. In this work, we report a rare case of left ventricular aneurysm associated with tricuspid valve avulsion in a 3-year-old boy after a road traffic accident. Our initial findings were a femur fracture and tricuspid valve avulsion. After 6 months transthoracic echocardiography revealed a giant ventricular aneurysm. Following that, the patient underwent ventricular aneurysmectomy, ventricular aneurysmosurgery, and tricuspid valvuloplasty with successful clinical outcomes.

## Case presentation

**Figure 1:**
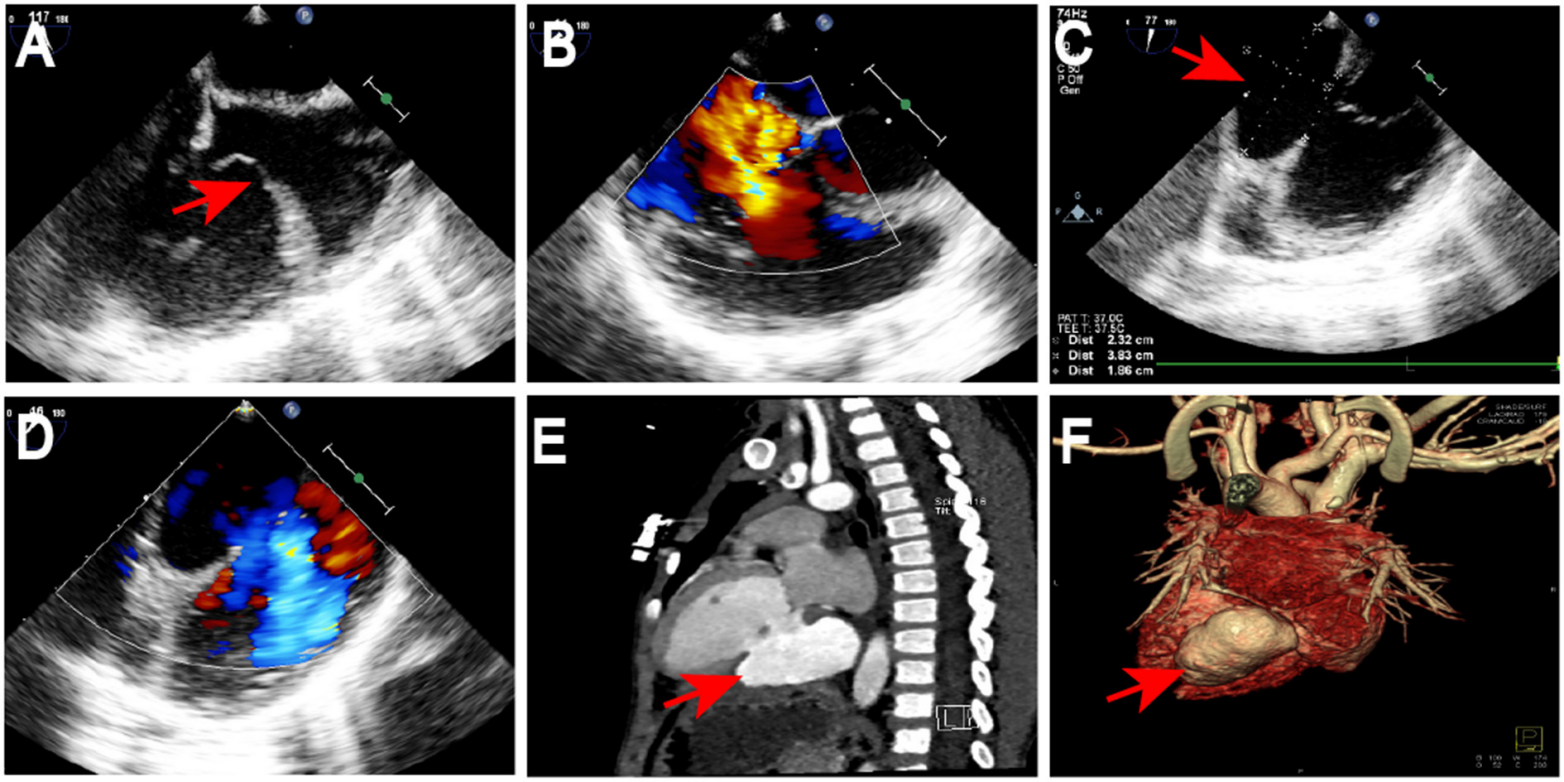
Preoperative echocardiography and computed tomography results. Anterior tricuspid leaflet prolapse was observed by TTE (red arrow; **A**). Tricuspid prolapse resulted in moderate tricuspid regurgitation **(B)**. TTE revealed avulsion of anterior tricuspid leaflet resulting in moderate tricuspid regurgitation, a 23 mm × 38 mm giant aneurysm with a 19-mm diameter of the neck in the LVPW (red arrow; **C**) and visible blood flow into the aneurysm **(D)**. CT scan and reconstruction confirmed a massive ventricular aneurysm in the LVPW (red arrow; **E,F**). TTE, Transthoracic Echocardiography; LVPW, left ventricular posterior wall; CT, computed tomography.

A 3-year-old child was admitted to our department with blunt injuries from a road traffic accident. Following the crash, he remained conscious while crying and was taken to our the emergency department of our hospital. The child felt considerable pain when pressure was applied to his right thigh during the physical examination without revealing any further abnormalities. Radiographs of his extremities showed a fractured right femur, and chest X-rays showed that the lungs were normal. An electrocardiogram (ECG) revealed low and flat T waves in leads III, aVF, and V1. Laboratory examination indicateda slight elevation in troponin to 21 ng/mL. Using transthoracic echocardiography (TTE), anterior tricuspid leaflet prolapse was observed because it is the first-line diagnostic tool for evaluating the tricuspid valve (Figure 1A). This tricuspid prolapse resulted in moderate tricuspid regurgitation (Figure 1B). The fracture was treated with a plaster cast in the Surgery department. The patient was then referred to our department for further treatment and was discharged after 1 week.

After 6 months, ECG revealed continuous abnormal Q waves in leads III and aVF during normal myocardial enzymes. TTE revealed avulsion of anterior tricuspid leaflet resulting in moderate tricuspid regurgitation, a 23 × 38 mm giant aneurysm with a 19-mm diameter of the neck in the left ventricular posterior wall (LVPW) (Figure 1C), and visible blood flow into the aneurysm (Figure 1D). Computed tomography (CT) scan and reconstruction confirmed a massive ventricular aneurysm in LVPW (Figures 1E,F). Finally, the patient was scheduled for surgery.

**Figure 2:**
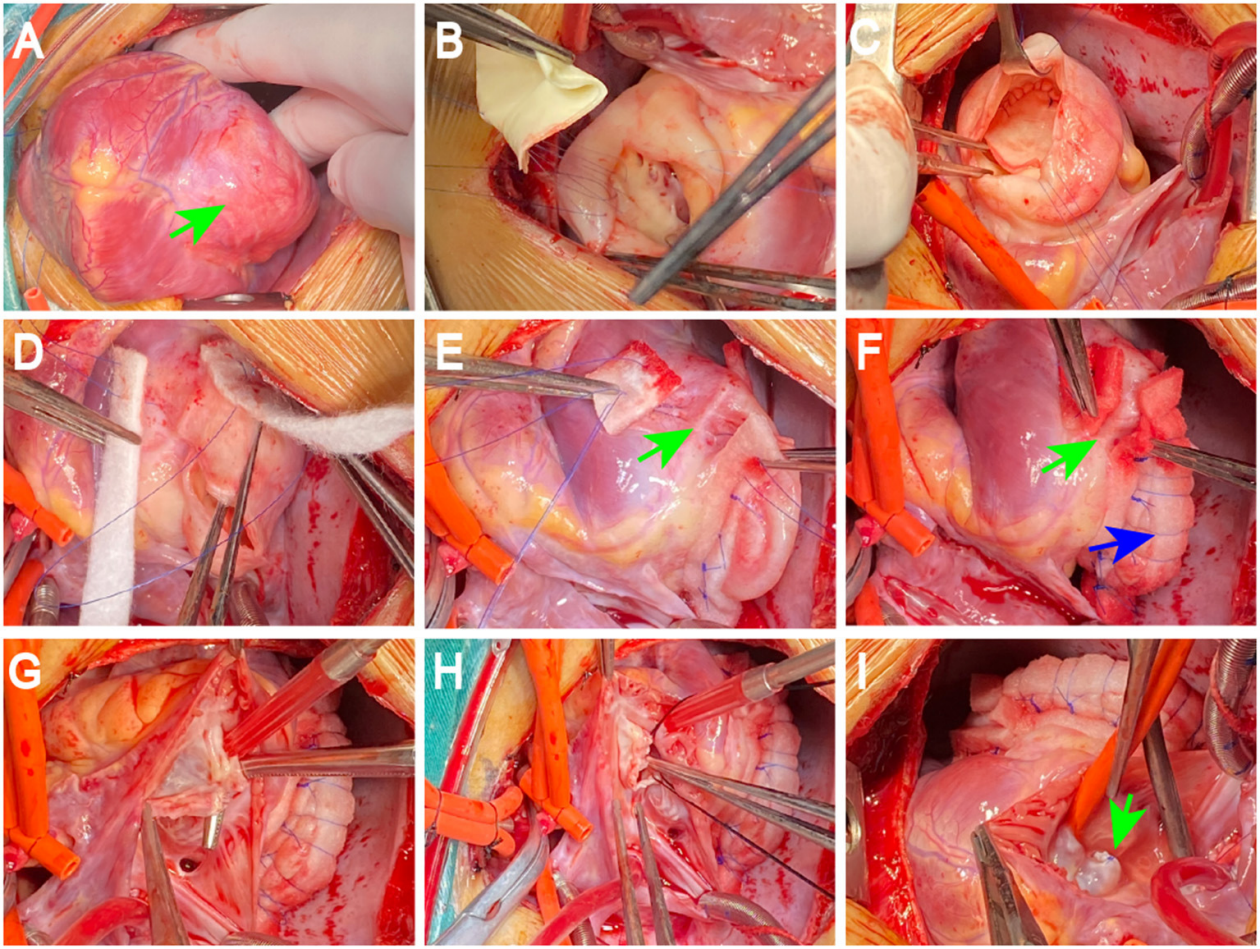
Surgical procedure. The aneurysmal portion of the left ventricle was thinned and was significantly expansive, the aneurysm well has sharply defined edges (blue arrow; **A**). The bovine pericardium was used to close the aneurysm's neck after being surgically incised **(B,C)**. Felt pads were used to close the epicardium over the patch **(D)**, resulting in a detour formed around the posterior descending artery (PDA) and PDA was well protected in a “sandwich” (green arrow; **E,F**). The third strip of the felt pad reinforced the “sandwich” structure (blue arrow; **F**). 5.0 Prolene suture lines were used to sew the rupture, valvuloplasty of the anterior tricuspid leaflet was performed **(G,H)**. Testing with saline solution injection showed no tricuspid regurgitation (green arrow; **I**). PDA: the posterior descending artery.

**Figure 3:**
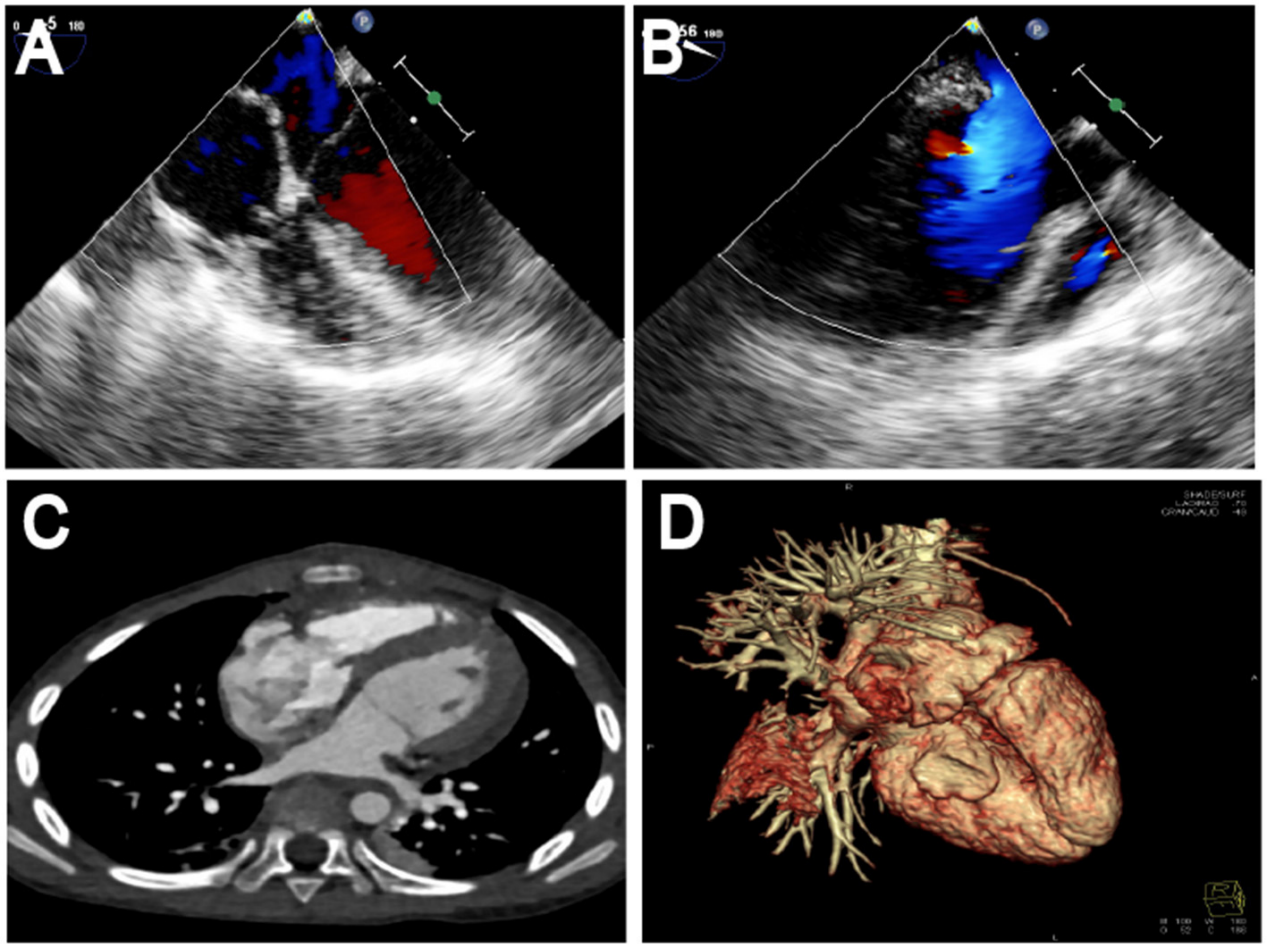
Follow-up. It showed no tricuspid regurgitation by intraoperative esophageal echocardiography showed no tricuspid regurgitation **(A)** and revealed no residual shunt for LVPW **(B)**. The postoperative CT scan and reconstruction revealed successful surgery **(C,D)**. LVPW, left ventricular posterior wall; CT, computed tomography.

**Figure 4:**
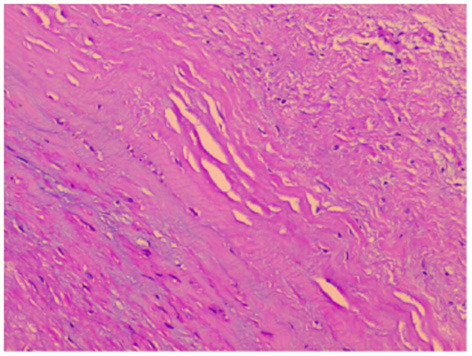
Histological examination. Histological examination revealed massive fibrous tissue in the aneurysm wall (Figure ×200).

During surgery, after sternotomy and the pericardium being incised, we found that the aneurysmal portion of the left ventricle was thinned and was greatly expanded (Figure 2A), and there was no pericardial adhesion. The myocardial layers were continuous, the aneurysm well had sharply defined edges (Figure 2A). After being surgically incised, the bovine pericardium was used to close the aneurysm's neck (Figures 2B,C). Felt pads were then employed to close the epicardium over the patch (Figure 2D), resulting in a detour formed around the posterior descending artery (Figures 2E,F). The third strip of the felt pad reinforced the “sandwich” structure (Figure 2F). A right atriotomy was performed to repair the anterior leaflet of the tricuspid valves with a 5 mm tear. 5.0 Prolene suture lines were utilized to sew the rupture, and valvuloplasty of the anterior tricuspid leaflet was performed (Figures 2G–I). 5.0 Prolene suture was used to figure-of-eight suture on the junction between the anterior annulus and the posterior annulus, and the junction between posterior annulus and septum annulus. Testing with saline solution injection and intraoperative esophageal echocardiography showed no tricuspid regurgitation (Figures 2I, 3A) and revealed no residual shunt for LVPW (Figure 3B). Histological examination demonstrated massive fibrous tissue in the aneurysm wall,myocardial fiber disappears is replaced by hyperplained fibrous tissue, fibrous connective tissue increases, and glass -like changes in local fiber tissue (uniform consistency, no structure and translucent protein accumulation) (Figure 4, ×200), consistent with trauma-induced aneurysms. The sketch of ventricular aneurysmorrhaphy and tricuspid valvuloplasty was provided (Supplementary Figures S1, S2). The postoperative CT scan and reconstruction revealed successful surgery (Figures 3C,D). One week after surgery, the patient was discharged, and follow-up care was provided in the outpatient setting. Finally, the child recovered well and was followed up for 12 months.

## Discussion

The case reported here has the following two notable features. Initially, the patient presented with only an anterior tricuspid leaflet avulsion on an ECG following blunt chest injury, but 6 months later he developed a massive ventricular aneurysm. A second observation was that the posterior left ventricle branch was well protected in the process of ventricular aneurysmectomy and aneurysmorrhaphy in a “sandwich” structure. In addition, the third strip of the felt pad was added to reinforce the “sandwich” structure.

The left ventricular aneurysm can be congenital or acquired, while both types present as a nodular structure protruding out of the cardiac chamber with regional ventricular wall expansion and thinning. A congenital ventricular aneurysm is a highly rare dysplastic heart malformation reported in around 0.04% of the population and is characterized by aberrant wall motion abnormality and large variations in size. Acongenital ventricular aneurysm is mainly presented in the neonatal period, and is generally accompanied by other congenital malformations, such as ventricular septal defect, atrial septal defect, tricuspid atresia, and tetralogy of Fallot (1). The acquired ventricular aneurysm is the most common in myocardial infarction, and it is also seen in cases such as cardiomyopathy (3), sarcoidosis (4), myocarditis (5), cardiovascular procedure complications (6), and blunt trauma. Traumatic ventricular aneurysms are extremely rare among them. The traumatic ventricular aneurysm has been reported in children under 2 9 years old (7–9). In these cases, blunt injuries were involved, such as chest injuries from vehicle crashes and falls from height. Several studies have reported that acquired traumatic ventricular aneurysms can be accompanied by ventricular septal perforation and hemopericardium.

Early findings in the present case included low T waves in leads III, aVF, V1, elevation in troponin, moderate tricuspid regurgitation, and no evidence of ventricular aneurysms. After 6 months of injury, continuous abnormal Q waves in leads III and aVF were demonstrated on ECG while myocardial enzymes were normal. Furthermore, TTE identified a massive aneurysm located in the left ventricle. This suggests that in pediatric cases suffering from a blunt chest injury, especially those experiencing cardiac injury such as complex valvular injury, the possibility of ventricular aneurysm should be considered.

An untreated large ventricular aneurysm carries a high risk of rupture and embolization. Due to the childbeing young and having a poor prognosis, conservative treatment arrangements were made to address the initial finding of tricuspid regurgitation. Surgery was performed after 6 months for the diagnosed sizeable ventricular aneurysm. The timing of surgery for traumatic ventricular aneurysms remains undetermined because of its rarity and lack of evidence regarding its management. In the present case, the ventricular aneurysm boundary and the scar wereformed after 6 months of injury, which may facilitatethe surgical procedure.

Traumatic tricuspid insufficiency is an uncommon complication of blunt cardiac injury (9). In avoidance of delayed right ventricular deterioration, early identification and surgical treatment of this disease are significant. A previous case of a 9-year-old boy exhibiting traumatic ventricular aneurysm combined with tricuspid insufficiency due to rupture of papillary muscles was eventually treated by valve replacement (10). For the present 3-year-old child presenting with femur fracture and tricuspid leaflet avulsion, valvuloplasty was performed with a satisfactory outcome at a 1-year follow-up.

Surgery for ventricular aneurysms requires protection for the major coronary branches, as the deformation and tamponade of the coronary postoperatively can cause malignant arrhythmia and death (11). As observed in this case, the ventricular aneurysm was located in the posterior wall close to the branches of the left ventricle. Resection was performed, and a bovine pericardium was used. Meanwhile, the pericardium was closed over the patch using two strips of felt pads (~0.8 × 4 cm) to form a “sandwich” structure with the left strip broken to avoid pressure on the posterior branch artery. Furthermore, the third strip of the felt pad was used to reinforce the “sandwich” structure to prevent hemorrhage, which is the first case using this way (a third strip) that has never been reported.

## Summary

We reported an extremely rare case of traumatic left ventricular aneurysm combining tricuspid valve avulsion following blunt injury. We present confirmed diagnosis, ventricular aneurysmorrhaphy, and tricuspid valvuloplasty with a successful clinical outcome. It suggests that in pediatric cases suffering from a blunt chest injury, especially experiencing a cardiac injury, a ventricular aneurysm may be considered and coronary arterial branches should be well protected in ventricular aneurysmectomy in a “sandwich.”

## Data availability statement

The original contributions presented in the study are included in the article/Supplementary material, further inquiries can be directed to the corresponding author.

## Ethics statement

Ethical review and approval was not required for the study on human participants in accordance with the local legislation and institutional requirements. Written informed consent to participate in this study was provided by the participants' legal guardian/next of kin. Written informed consent was obtained from the minor(s)' legal guardian/next of kin for the publication of any potentially identifiable images or data included in this article.

## Author contributions

DZ and SW performed the data analyses and wrote the manuscript. WT contributed to provided and analyzed intraoperative and postoperative cardiac ultrasound images required for the study. RX and YL contributed significantly to analysis and manuscript preparation. DL helped perform the analysis with constructive discussions. All authors contributed to the article and approved the submitted version.

## Funding

This work was supported by the National Natural Science Foundation of China (Grant No. 82160060) and Guizhou Provincial Science and Technology Projects ZK [2022] YB669 and ZK [2022] YB652.

## Conflict of interest

The authors declare that the research was conducted in the absence of any commercial or financial relationships that could be construed as a potential conflict of interest.

## Publisher's note

All claims expressed in this article are solely those of the authors and do not necessarily represent those of their affiliated organizations, or those of the publisher, the editors and the reviewers. Any product that may be evaluated in this article, or claim that may be made by its manufacturer, is not guaranteed or endorsed by the publisher.
