# Supplementary figures and images for: Case report: Traumatic ventricular aneurysm combining tricuspid valve avulsion in a child: Diagnostic findings and treatment protocols
Source: Front Cardiovasc Med. 2022 Aug 23;9:928265. doi: 10.3389/fcvm.2022.928265 (PMC9445196; doi:10.3389/fcvm.2022.928265)

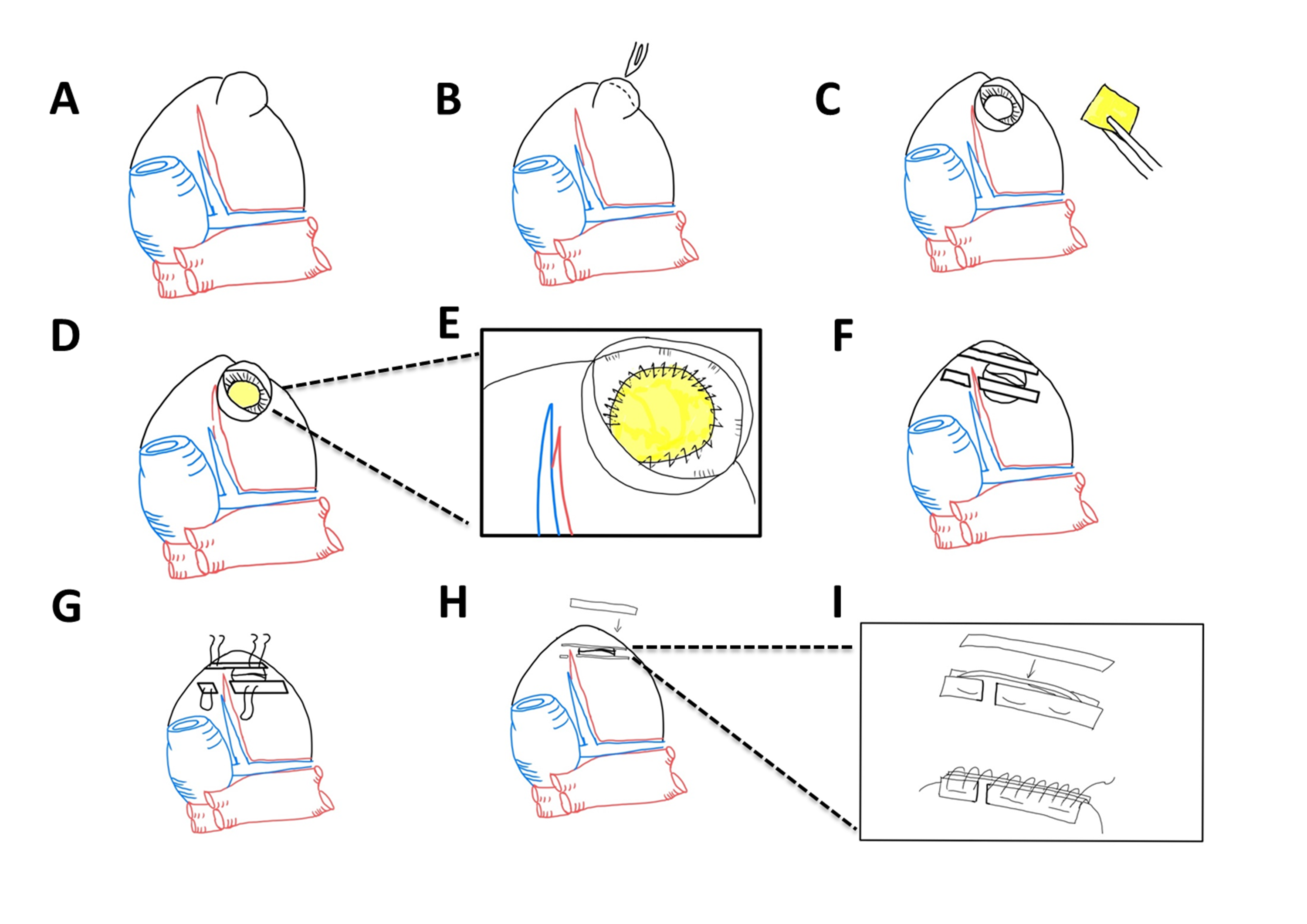

Supplement: Supplementary file 1 [file Image_1.tif]

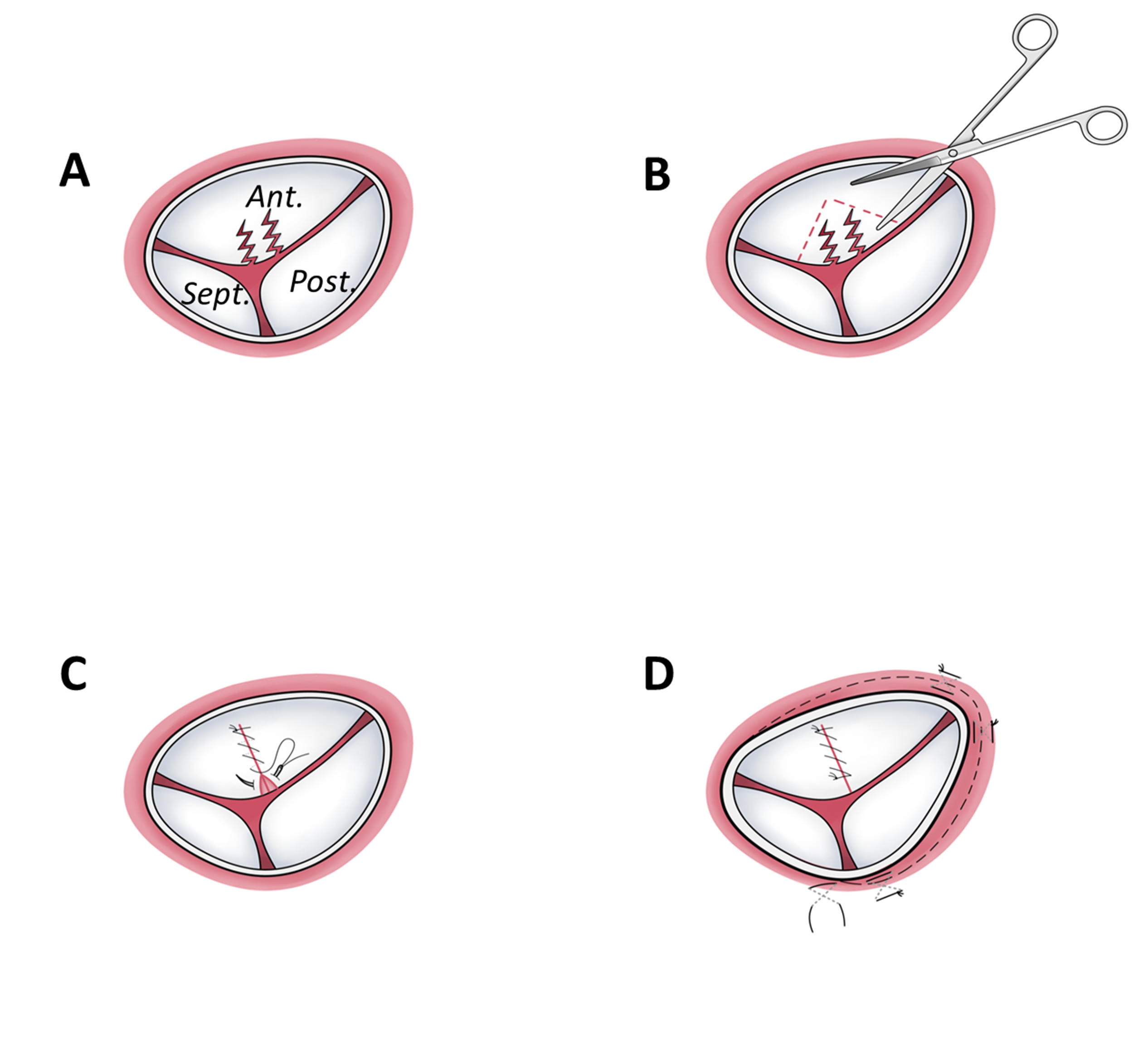

Supplement: Supplementary file 2 [file Image_2.tif]
